# Change in Dynamic Hyperinflation After Bronchoscopic Lung Volume Reduction in Patients with Emphysema

**DOI:** 10.1007/s00408-020-00382-x

**Published:** 2020-07-24

**Authors:** Marlies van Dijk, Karin Klooster, Jorine E. Hartman, Nick H. T. ten Hacken, Dirk-Jan Slebos

**Affiliations:** 1 Department of Pulmonary Diseases, University Medical Center Groningen, Research Institute for Asthma and COPD Groningen, University of Groningen, Groningen, The Netherlands; 2 Department of Pulmonary Diseases, AA11, University Medical Center Groningen, PO Box 30001, 9700 RB Groningen, The Netherlands

**Keywords:** COPD, Emphysema, Dynamic hyperinflation, Bronchoscopic lung volume reduction

## Abstract

**Background and Purpose:**

In patients with severe emphysema, dynamic hyperinflation is superimposed on top of already existing static hyperinflation. Static hyperinflation reduces significantly after bronchoscopic lung volume reduction (BLVR). In this study, we investigated the effect of BLVR compared to standard of care (SoC) on dynamic hyperinflation.

**Methods:**

Dynamic hyperinflation was induced by a manually paced tachypnea test (MPT) and was defined by change in inspiratory capacity (IC) measured before and after MPT. Static and dynamic hyperinflation measurements were performed both at baseline and 6 months after BLVR with endobronchial valves or coils (treatment group) or SoC (control group).

**Results:**

Eighteen patients underwent BLVR (78% female, 57 (43–67) years, FEV_1_ 25(18–37) %predicted, residual volume 231 (182–376) %predicted). Thirteen patients received SoC (100% female, 59 (44–74) years, FEV_1_ 25 (19–37) %predicted, residual volume 225 (152–279) %predicted. The 6 months median change in dynamic hyperinflation in the treatment group was: + 225 ml (range − 113 to + 803) (*p* < 0.01) vs 0 ml (− 1067 to + 500) in the control group (*p* = 0.422). An increase in dynamic hyperinflation was significantly associated with a decrease in residual volume (*r* = − 0.439, *p* < 0.01).

**Conclusion:**

Bronchoscopic lung volume reduction increases the ability for dynamic hyperinflation in patients with severe emphysema. We propose this is a consequence of improved static hyperinflation.

## Introduction

In patients with severe emphysema chronic inflammation results in airway and lung parenchyma damage which is associated with reduced lung elastic recoil and increased airway resistance [1]. The combination of reduced elastic recoil and increased airway resistance can lead to a progressive increase of residual volume (RV) and end-expiratory lung volume (EELV), called static hyperinflation [1]. Increased hyperinflation can lead to dyspnea and consequently to reduced exercise capacity and poor quality of life [2]. Apart from static hyperinflation, exercise can lead to an additional increase in hyperinflation and a further decrease of the inspiratory capacity [1, 2]. This is called dynamic hyperinflation, which is superimposed on top of static hyperinflation. In patients with severe emphysema and severe static hyperinflation bronchoscopic lung volume reduction (BLVR) with endobronchial valves (EBV) or coils can lead to a statistically significant and clinically relevant reduction of static hyperinflation [3]. Furthermore, an improvement of dynamic hyperinflation has been demonstrated in a small group of patients after lung volume reduction surgery [4]. On the other hand, it could also be hypothesized that the improvement of static hyperinflation after bronchoscopic lung volume reduction leads to a larger rest inspiratory capacity (IC), leaving more room for dynamic hyperinflation to occur.

For this study our aim was to investigate if (and if so how) dynamic hyperinflation changed after bronchoscopic lung volume reduction compared to standard of care in patients with severe emphysema and severe static hyperinflation. Additionally, we aimed to investigate if there was an association between change in dynamic hyperinflation and change in parameters reflecting static hyperinflation and exercise tolerance.

## Methods

### Study Design and Population

This was a single-center prospective cohort study in patients with severe emphysema who underwent a bronchoscopic lung volume reduction (BLVR) treatment with either endobronchial valves or coils or standard of care (SoC, no treatment) at the pulmonary department of the University Medical Center Groningen, the Netherlands. All subjects were clinically stable, on optimal medication and had stopped smoking at least 6 months before the study. All subjects participated in one of our bronchoscopic lung volume reduction trials (Clinical trial identifiers: NCT01421082; NCT01101958; NTR2876), which were approved by the local ethics committee. All subjects gave written informed consent. All subjects were included between June 2011 and July 2012. The baseline assessment measurements of this study population were part of an earlier publication [5]. From this baseline cohort patients were randomly invited for follow-up measurements for this study.

### Measurements

All measurements were performed at baseline and 6 months after BLVR treatment or SoC.

Subjects were instructed to use their regular inhalation medication. An additional 400 µg of salbutamol was administered 15 min before the pulmonary function measurements. Spirometry, body plethysmography and diffusion capacity were measured using the Jaeger MasterScreen™ Body plethysmograph (CareFusion, Germany) and were performed according to the ATS/ERS guidelines using the reference values from the European Community for Coal and Steel [6–8]. The 6-min walk test (6MWT) was performed according to ATS recommendations [9]. The St. George’s Respiratory Questionnaire (SGRQ), and the modified Medical Research Council dyspnea scale (mMRC) were used to measure quality of life and dyspnea severity, respectively [10, 11].

**Fig. 1:**
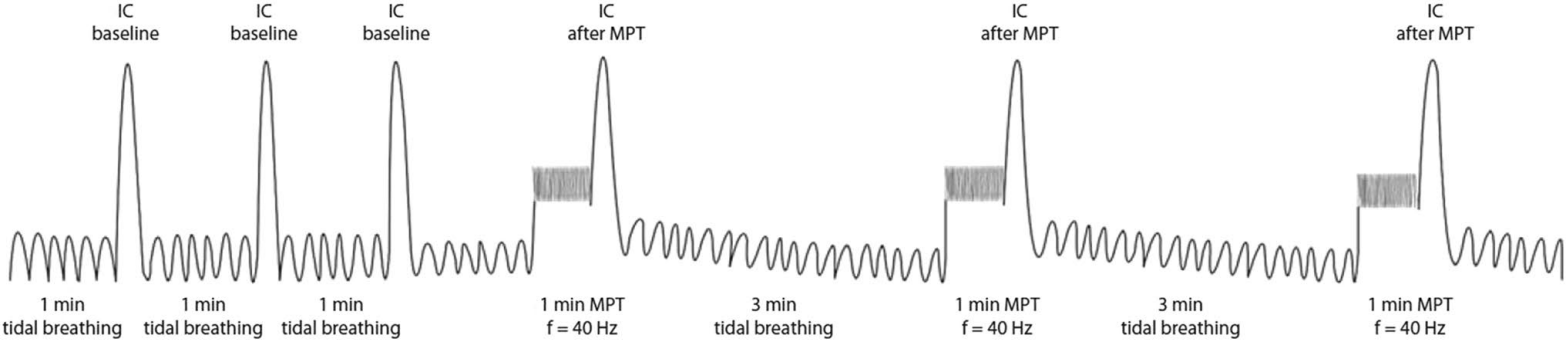
Schematic overview of the dynamic hyperinflation measurement. *IC* inspiratory capacity, *MPT* manually paced tachypnea, *f* frequency (40 times/min). Image reprinted with permission of respiration [5]

Dynamic hyperinflation was measured using a manually paced tachypnea (MPT) test using the breath-by-breath method (Oxycon Pro™, CareFusion, Germany) during a 15-min protocol (See Fig. 1 for a schematic overview of the MPT procedure) [5]. During tidal breathing the subjects were asked to perform a minimum of 3 slow maximum inspirations (IC maneuver) with 1 min of normal tidal breathing between each maneuver. After this, the technician asked the subject to increase their breathing frequency (BF) to a rate of 40 times per min for 1 min. The technician used a visual real-time registration of the BF and provided the subject with vocal feedback of their BF. After 1 min of tachypnea, the subjects immediately performed an IC maneuver. The MPT procedure was repeated at least 3 times, with 3 min of normal tidal breathing between maneuvers. To establish the baseline IC (IC_baseline_), we calculated the mean value of 3 reproducible IC’s (within 150 ml). To establish the IC post tachypnea (IC_MPT_) we calculated the mean value of the 2 highest and reproducible IC’s (within 150 ml).

### Statistics

Power was calculated based on mean change in IC of 0.5L (SD 0.4) [12]. With a power of 0.80 and alpha of 0.05 at least 12 patients per group needed to be included. Data was calculated as median (minimum–maximum) unless indicated otherwise. Dynamic hyperinflation was calculated by the absolute change in IC (IC_MPT_ minus IC_baseline_). A negative value of the absolute change in IC indicates a greater amount of dynamic hyperinflation.

A Mann–Whitney U test was performed to compare baseline and follow-up lung function parameters, SGRQ and 6MWD. A Wilcoxon signed ranktest was used to compare baseline characteristics, change in lung function parameters, SGRQ and 6MWD between groups (BLVR vs. SoC). A Spearman correlation coefficient was calculated to assess the association between change in dynamic hyperinflation and change in static hyperinflation, airflow obstruction and 6-min walk distance. A *p*-value of < 0.05 was considered statistically significant. IBM SPSS Statistics version 23 (IBM, Armonk, NY, USA) was used for all analyses.

## Results

We studied 31 clinically stable patients with severe emphysema. Thirteen patients received SoC (100% female, 59 (44–74) years, FEV_1_ 25 (19–37) %predicted, residual volume 225 (152–279) %predicted. Eighteen patients underwent BLVR (78% female, 57 (43–67) years, FEV_1_ 25(18–37) %predicted, residual volume 231 (182–376) %predicted. Of these, ten patients were treated with coils, eight patients received endobronchial valves. There were no statistically significant differences in baseline characteristics between the control and treatment group (Table 1).

**Table 1:** Baseline characteristics

Baseline characteristic	Treatment group *n* = 18	Control group *n* = 13	*p*-value
Female—no. (%)	14 (78%)	13 (100%)	0.073
Age—year	57 (43 to 67)	59 (44 to 74)	0.40
Body-mass index—kg/m^2^	23 (16 to 29)	22 (18 to 26)	0.32
Cigarette smoking—no. of pack years	38 (5 to 80)	40 (23 to 110)	0.95
FEV_1_			
Liters	0.63 (0.45 to 1.01)	0.69 (0.40 to 0.87)	0.33
% of predicted	25 (18 to 37)	25 (19 to 37)	0.56
FVC			
Liters	2.38 (1.28 to 3.71)	2.01 (1.08 to 2.92)	0.11
% of predicted	70 (44 to 101)	63 (50 to 113)	0.48
RV			
Liters	4.87 (2.93 to 7.71)	4.10 (3.09 to 5.58)	0.11
% of predicted	231 (182 to 376)	225 (152 to 279)	0.24
TLC			
Liters	7.48 (5.75 to 10.76)	6.83 (5.27 to 7.92)	0.08
% of predicted	134 (120 to 183)	135 (114 to 150)	0.56
Ratio of RV to TLC—%	65 (48 to 74)	65 (52 to 75)	0.97
Ratio of IC to TLC—%	20 (16 to 38)	24 (16 to 37)	0.38
R_AW_			
kPa*S/L	0.76 (0.33 to 1.21)	0.67 (0.47 to 1.00)	0.98
% of predicted	252 (109 to 404)	225 (158 to 334)	0.98
Dynamic hyperinflation—ml	− 610 (− 1240 to − 120)	− 608 (− 1260 to − 260)	0.90
Carbon monoxide diffusing capacity			
mmol/(min*kPa)	3.12 (1.93 to 5.52)	2.76 (1.05 to 4.35)	0.37
% of predicted	32 (24 to 69)	35 (14 to 57)	0.96
Arterial blood gas (on room air)— kPa			
PaO_2_	9.2 (7.1 to 11.9)	8.5 (7.6 to 12.6)	0.32
PaCO_2_	5.4 (4.4 to 6.9)	5.2 (4.2 to 6.6)	0.41
6-min walk test			
Distance—meters	318 (160 to 485)	400 (160 to 459)	0.17
Questionnaires			
SGRQ total score—points	60 (25 to 79)	59 (43 to 89)	0.75
mMRC—points	3 (1 to 4)	3 (2 to 4)	0.83

Data is represented as median (min to max) or number (%)

There were no statistically significant differences between the treatment group and control group (Mann–Whitney U test)

*FEV*_*1*_ forced expiratory volume in 1 s, *FVC* forced vital capacity, *RV* residual volume, *TLC* total lung capacity, *R*_*aw*_ airway resistance, *SGRQ* St George Respiratory Questionnaire, *CCQ* Clinical COPD Questionnaire

**Fig. 2:**
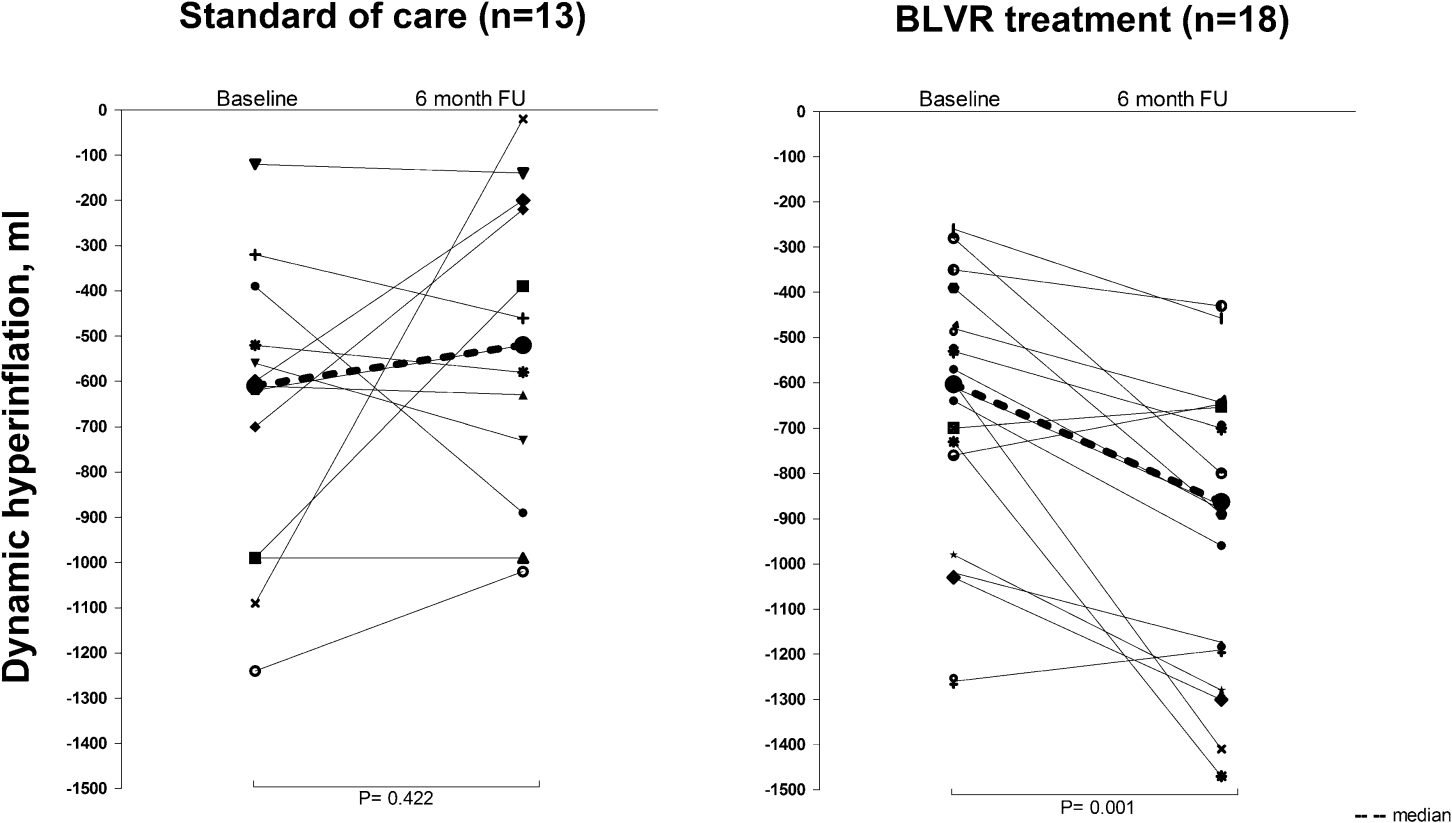
Individual outcomes of dynamic hyperinflation at baseline and 6 months follow-up. *BLVR* bronchoscopic lung volume reduction. De dotted line reflects the difference between median dynamic hyperinflation at baseline and median dynamic hyperinflation at follow-up

Dynamic hyperinflation changed significantly with − 225 ml (− 803 to + 113) (*p* < 0.01) 6 months after BLVR. In the group of subjects receiving SoC, there was no significant change in dynamic hyperinflation (0 ml, range − 1067 to + 500). See Fig. 2 for individual outcomes. There were no statistically significant differences in change in dynamic hyperinflation between subjects who were treated with endobronchial valves [− 232 ml (− 803 to + 77)] and subjects who were treated with coils [− 170 ml (− 517 to + 113)].

In the treated subjects (*n* = 18), there were statistically significant improvements in FEV_1_, residual volume, and SGRQ total score compared to baseline (all *p* < 0.01), which were not present in the SoC group. The between-group differences were all significantly different (Table 2).

**Table 2:** Median change in lung function, dynamic hyperinflation, 6MWD and SGRQ 6 months after BLVR treatment (*n* = 18) or SoC (*n* = 13)

	BLVR treatment (*n* = 18)	Standard of care (*n* = 13)	BLVR vs. SoC *p*-value
ΔDH			0.002
ml	− 225 (− 803 to + 113)*	0 (− 500 to + 1067)	
Relative change (%)	− 33 (− 186 to + 15)	0 (− 128 to + 988)	
ΔFEV_1_			0.034
ml	+ 110 (− 130 to + 770)*	+ 20 (− 10 to + 13)	
Relative change (%)	+ 22 (− 16 to + 76)	+ 3 (− 13 to 17)	
∆IC (rest)			0.010
ml	+ 200 (− 350 to + 1530)*	− 33 (− 430 to + 270)	
Relative change (%)	+ 11 (− 12 to + 70)	-2 (− 23 to + 15)	
ΔRV			< 0.001
ml	− 765 (− 3010 to + 40) *	+ 40 (− 140 to + 280)	
Relative change (%)	− 15 (− 39 to + 1)	+ 1 (− 3 to 7)	
∆TLC			0.002
ml	− 295 (− 690 + 230) *	+ 40 (− 290 to + 260)	
Relative change (%)	− 295 (− 690 to + 230)	+ 0.6 (− 3.7 to + 3.7)	
∆Ratio of RV to TLC—%	− 8 (− 25 to + 1)*	+ 0 (− 2 to + 4)	< 0.001
∆Ratio of IC to TLC—%	+ 3 (− 3 to + 20)	− 1 (− 7 to + 4)	0.006
Δ*R*_aw_ (kPa*S/L)	− 0.14 (− 0.48 to + 0.29)*	0.01 (− 0.15 to + 0.29)	0.06
Δ6MWD meters	+ 55 (+ 8 to + 233) *	− 17 (− 134 to + 53)	< 0.001
ΔSGRQ points	− 11 (− 53 to + 6)*	− 1 (− 25 to + 9)	0.020

All changes between baseline and follow-up were statistically significant for the treatment group, *p < 0.05. There were no statistically significant changes between baseline and follow-up for the SoC group measured by Mann–Whitney U test

*BLVR* bronchoscopic lung volume reduction, *DH* dynamic hyperinflation, *FEV*_*1*_ forced expiratory volume in 1 s, *IC* inspiratory capacity, *RV* residual volume, *TLC* total lung capacity, *R*_*aw*_ airway resistance, *6MWD* 6-min walk distance, *SGRQ* St Georges Respiratory Questionnaire

**Fig. 3:**
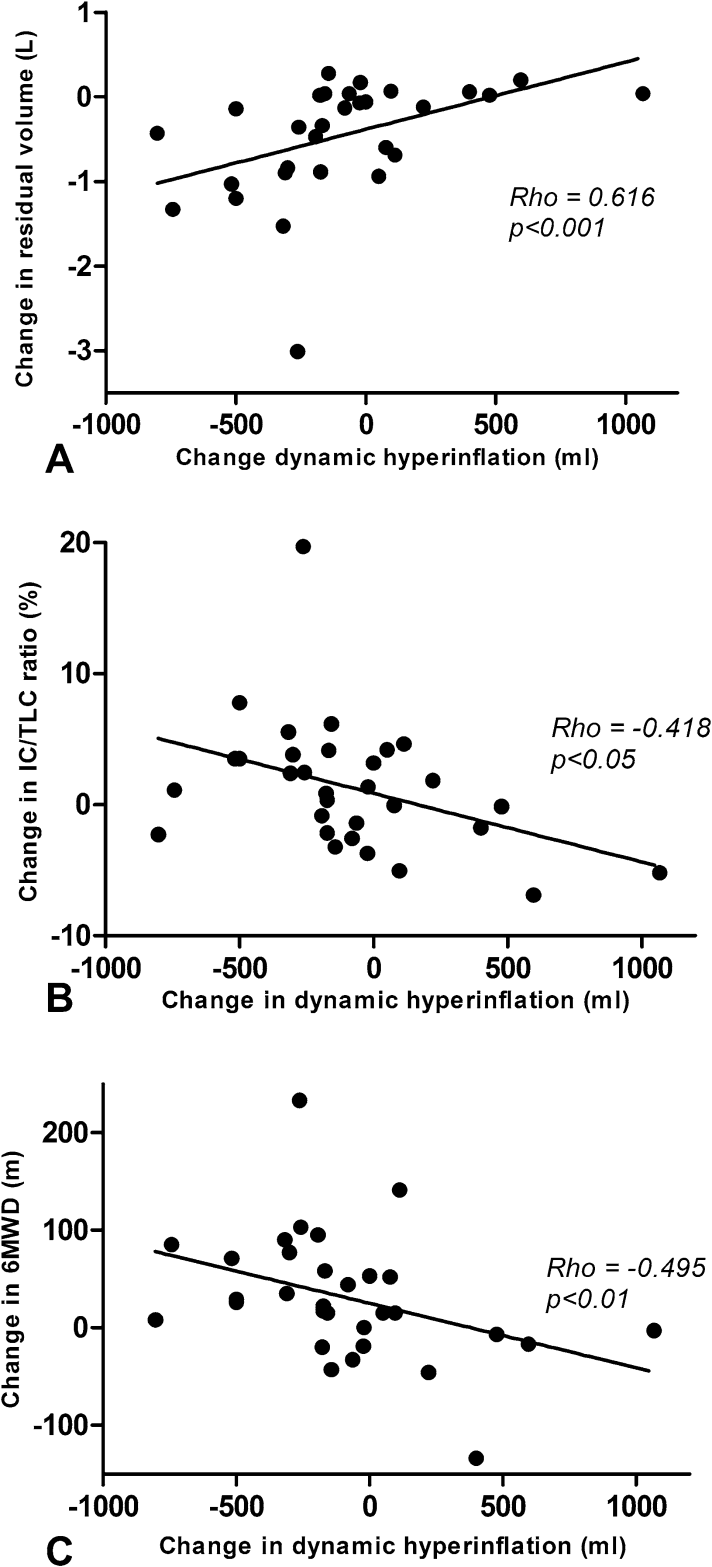
Association between change in dynamic hyperinflation and change in RV, IC/TLC and 6MWD. **a** Association between change in dynamic hyperinflation and change in residual volume. **b** Association between change in dynamic hyperinflation and change in IC/TLC ratio. **c** Association between change and 6MWD. *RV* residual volume; *IC* inspiratory capacity; *TLC* total lung capacity; *6MWD* 6-min walk distance

An increase in dynamic hyperinflation was significantly associated with a decrease in residual volume (rho = 0.616, *p* < 0.001), an increase in IC/TLC ratio (*r* = − 0.418, *p* < 0.05) and with an increase in 6MWD (*r* = − 0.495, *p* < 0.01) (see Fig. 3) for the treatment and control group combined.

## Discussion

In this single-center prospective cohort study, we investigated change in dynamic hyperinflation measured by a manually paced tachypnea test after lung volume reduction treatment with either endobronchial valves or coils compared to standard of care. We demonstrated a significant increase in dynamic hyperinflation after BLVR, which was not the case for standard of care. Change in dynamic hyperinflation showed a significant inverse association with change in residual volume and a significant association with change in IC/TLC ratio and change in 6-min walk distance.

Our group has previously shown that performing a manually paced tachypnea test is feasible and safe in patients with severe COPD [5]. Interestingly, this previous study showed a negative association between dynamic hyperinflation and the 6MWD, i.e., more severe dynamic hyperinflation was associated with a better exercise tolerance. This is in line with the results of the current study where a larger increase of dynamic hyperinflation was associated with a larger increase in 6MWD. This may seem contrary to expectations, since dynamic hyperinflation is associated with a reduced exercise tolerance [13]. A possible explanation is that in this group of patients with severe static hyperinflation the inspiratory capacity is very low even in rest, and this leaves little space for dynamic hyperinflation to occur. When successful lung volume reduction treatment is performed and static hyperinflation decreases, the inspiratory capacity increases as does the ability to develop dynamic hyperinflation on tachypnea. Therefore, the increase in dynamic hyperinflation could even be seen as a positive marker of lung volume reduction treatment, since it indicates an improvement of the inspiratory capacity.

Contrary to our results, several other studies have demonstrated a reduction of dynamic hyperinflation after lung volume reduction treatment [4, 12, 14, 15]. However, it is difficult to compare these studies to our own results because there are some important differences. First of all, different techniques were used, i.e., measurement of inspiratory capacity during rest and cardiopulmonary exercise testing (CPET) [4, 12, 14] and optoelectronic plethysmography [15].

Secondly, the breathing frequency was lower in the other studies compared to this study (25–28 times/min versus 40 times/min). And, perhaps most importantly, different definitions for dynamic hyperinflation were used. We defined dynamic hyperinflation as the change in inspiratory capacity after a period of tachypnea compared to resting breathing frequency. However, if end-expiratory lung volume at the end of the test is used to define dynamic hyperinflation, this may lead to a different outcome, because this value is also influenced by a change in static hyperinflation. Severity of airflow obstruction and static hyperinflation were comparable to our subjects in all studies.

On a group level there was no change in dynamic hyperinflation in the control group after 6 months of standard of care. However, as shown in Fig. 2, on an individual level there were large variations in dynamic hyperinflation at baseline and follow-up. We propose that this is a reflection of real-life variability of dynamic hyperinflation in patients with COPD, most likely caused by changes in small airways disease such as mucous impaction and airway wall edema [13]. However, variability in the procedure can also play a role. Lahaije et al. found a repeatability coefficient of 8.5% for the MPT in patients with moderate COPD [16].

If dynamic hyperinflation increases after bronchoscopic lung volume reduction, does this have therapeutic consequences? We believe the most important message is to reinforce adequate breathing techniques in our patients, focusing on slow, deep breaths during exercise. A meta-analysis showed that long-acting bronchodilators did have an effect on EELV during exercise, but this was a consequence of an improved IC in rest (i.e., reduction in static hyperinflation) [17]. Interestingly, O’Donnel and colleagues demonstrated a protective effect of dynamic hyperinflation at lower exercise intensities by attenuation of the expiratory flow [18].

Our study does have some limitations. The group of subjects was relatively small. Especially since our results relating to dynamic hyperinflation are different from earlier studies, it would be interesting to investigate the change in dynamic hyperinflation by MPT in another, larger cohort of patients with COPD who undergo bronchoscopic lung volume reduction. Furthermore, the MPT test induces dynamic hyperinflation through tachypnea, but does not require exercise which is usually the trigger for DH to develop in patients with COPD. Excessive mechanical loading, ventilation of physiological dead space, arterial hypoxemia and early metabolic acidosis due to skeletal muscle deconditioning can lead to increased inspiratory neural drive to the respiratory muscles during exercise in COPD patients [19]. Furthermore, testing DH with CPET provides additional information on the influence of DH on exercise-induced dyspnea, cardiovascular function and muscle function [20]. A future study using both MPT and CPET to investigate change in dynamic hyperinflation would therefore be interesting.

## Conclusion

In our population of patients with severe emphysema, we found that dynamic hyperinflation increases after bronchoscopic lung volume reduction with coils or endobronchial valves, while static hyperinflation significantly improves. No significant changes were seen in the standard of care group. We propose that the underlying mechanism for this is that bronchoscopic lung volume reduction treatment improves static hyperinflation and therefore increases the ability for dynamic hyperinflation to occur in patients with severe emphysema.

## Data Availability

The datasets used and/or analyzed during the current study are available from the corresponding author on reasonable request.
